# Cellular Uptake and Delivery-Dependent Effects of Tb^3+^-Doped Hydroxyapatite Nanorods

**DOI:** 10.3390/molecules22071043

**Published:** 2017-06-23

**Authors:** Yan Wei, Ying He, Xiyu Li, Haifeng Chen, Xuliang Deng

**Affiliations:** 1 Department of Geriatric Dentistry, National Engineering Laboratory for Digital and Material Technology of Stomatology, Beijing Laboratory of Biomedical Materials, Peking University School and Hospital of Stomatology, Peking University, Beijing 100081, China; kqweiyan@126.com (Y.W.); kqheying@163.com (Y.H.); 2 Department of Biomedical Engineering, College of Engineering, Peking University, 5 Yiheyuan Road, Haidian District, 100871 Beijing, China; lixiyu@scu.edu.cn

**Keywords:** hydroxyapatite nanostructures, cellular uptake, tissue distribution, toxicity

## Abstract

With the increasing interest in hydroxyapatite (HA) nanostructures for use in biomedicine, the systematic evaluation of their potential effects on biological systems is becoming critically important. In this work, we report the in vitro cellular uptake, in vivo tissue distributions and toxicity of Tb^3+^-doped HA (HA-Tb) after short-, intermediate-, and long-term exposure. Transmission electron microscopy analysis indicated that HA-Tb was taken up by cells via vesicle endocytosis. Cell proliferation and cytotoxicity assay, combined with confocal laser scanning microscopy, indicated excellent cell viability with no changes in cell morphology at the examined doses. Three HA-Tb delivery methods (intraperitoneal, intragastric, and intravenous) resulted in similar time-dependent tissue distributions, while intraperitoneal injection produced the highest bioavailability. HA-Tb initially accumulated in livers and intestines of rats (4 h to one day after administration), then became increasingly distributed in the kidney and bladder (seven days), and finally decreased in all tissues after 30 to 90 days. No histopathological abnormalities or lesions related to treatment with HA-Tb were observed. These results suggest that HA-Tb has minimal in vitro and in vivo toxicity, regardless of the delivery mode, time, and dose. The findings provide a foundation for the design and development of HA for biological applications.

## 1. Introduction

Nanosized hydroxyapatite (HA), which possesses a high surface-to-volume ratio, high reactivity, and biomimetic morphology, has been proposed for a variety of biomedical applications, including drug delivery, gene transaction, cellular imaging, and biosensing [1,2,3,4]. HA-based nanomaterials show promise as scaffolds for controlled bone or dental regeneration [5,6,7,8]. Due to the interest in HA and HA-based nanomaterials for biomedical applications, their effects on biological systems have become a great concern. Buma et al. reported wear particles originating from HA-coated hip prosthesis in the macrophages of the intertrabecular medullary space [9,10]. Tamaki et al. found that wear particles originating from hip prosthesis enhanced the osteolytic potential of macrophages from bone marrow [11]. These findings support the hypothesis that HA is generally able to migrate away from the implantation site. However, other questions remain. If HA particles can migrate through the cell linings at the absorption site, how fast do they move through the body? Does the distribution of HA particles vary with the route of exposure? Are HA particles distributed evenly among all organs and tissues? These questions have great theoretical and clinical relevance for the safe and efficient application of HAs in biomedical applications.

To understand the biological behaviors of nanomaterials, it is imperative to design suitable tracing techniques for efficient and accurate assays. Conventional histopathological observation using immunofluorescent staining is not suitable for long-term tracking because of fluorescence quenching [12]. In contrast, radioactive elements such as ^125^I, ^177^Lu, ^166^Ho, ^175^Yb [13], and quantum dots, such as Cd, Se, Te, Hg, and Pb [14] are able to macroscopically label and visualize tissue distribution; however, their radioactivity and dose-dependence cytotoxicity inevitably increase the risk of adverse reactions. Based on their low phonon energy and, thus, the minimum quenching of emissive Ln^3+^ ions, lanthanide salts have been proposed as suitable host materials for luminescent probes in biomedical research [15,16,17]. In particular, Tb^3+^ ions have been found to possess strong luminescence and emission within the visible wavelength range. Tb^3+^ ions also have 5D0 luminescence lifetimes in the μs and ms range and are nanoscale and non-toxic.

Herein, we propose Tb^3+^ doping as a tracking technique for analyzing the biological response to HA nanomaterials. To test the validity and safety of Tb^3+^-doped HA (HA-Tb) nanostructures for potential bio-applications, we report the biological compatibility and effects of HA-Tb, including in vitro cellular uptake, in vivo delivery-dependent tissue distribution, and toxicity. 

## 2. Results

### 2.1. Characterization of HA-Tb Nanorods

**Figure 1:**
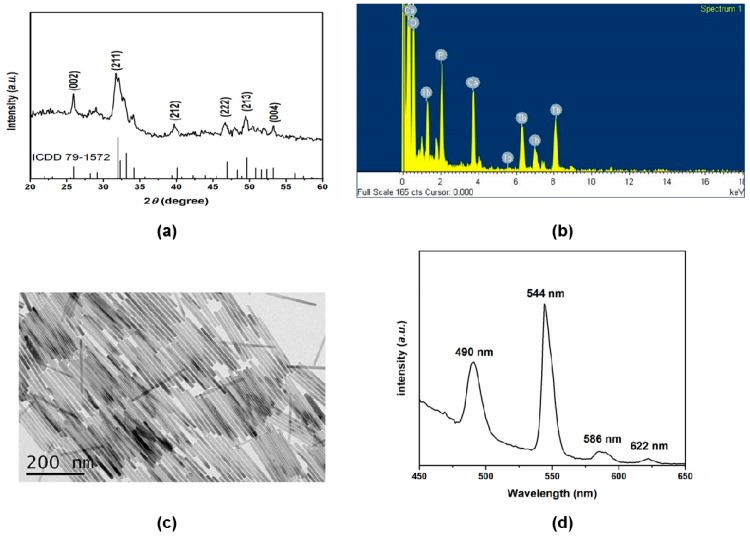
Characterization of Tb^3+^-doped hydroxyapatite (HA-Tb) nanorods: (**a**) X-ray diffraction pattern of HA-Tb nanorods after hydrothermal synthesis; (**b**) elemental mapping of HA-Tb nanorods by transmission electron microscopy (TEM) with energy-dispersive spectroscopy; (**c**) representative TEM image showing the nanorod morphology; and (**d**) luminescence spectrum of HA-Tb crystals.

To characterize HA-Tb nanorods, powder X-ray diffraction (XRD), transmission electron microscopy (TEM), with energy-dispersive spectroscopy (EDS) and luminescence spectrophotometry, were employed to examine the crystal structure, morphology, and upconversion properties, respectively. Figure 1a shows the XRD pattern of the synthesized HA nanorods doped with Tb^3+^ ions. All diffraction peaks were indexed to the typical hexagonal phase of HA (ICDD 79-1572). The sharp characteristic diffraction peaks at approximately 25.9°, 31.7°, 32.1°, 39.7°, and 46.7° correspond to the (002), (211), (212), (222), and (213) lattice planes of the classic hexagonal phase of HA, respectively. No peaks of impurity phases were identified in the XRD pattern of the HA-Tb nanorods, implying that the doped Tb^3+^ ions were incorporated into the HA lattice. The TEM-EDS element mapping of the crystals also demonstrated that the Tb^3+^ ions were successfully incorporated into the crystal structure (Figure 1b). The TEM image in Figure 1c shows straight, rod-like HA-Tb nanorods with lengths ranging from 217 to 340 nm. These results demonstrate that the slender HA-Tb single crystals exhibited a relatively uniform morphology and narrow size distribution. The luminescence spectrum of the HA-Tb crystals (Figure 1d, excitation at 280 nm) shows peaks at 490 nm (5D4–7F6), 544 nm (5D4–7F5), 586 nm (5D4–7F4), and 622 nm (5D4–7F3). Thus, the hydrothermal method employed in this study successfully produced Tb^3+^-doped upconversion HA nanorods with excellent crystallinity, uniform morphology and prominent luminescence.

### 2.2. Interaction between HA-Tb Nanorods and MC3T3-E1 Cells

**Figure 2:**
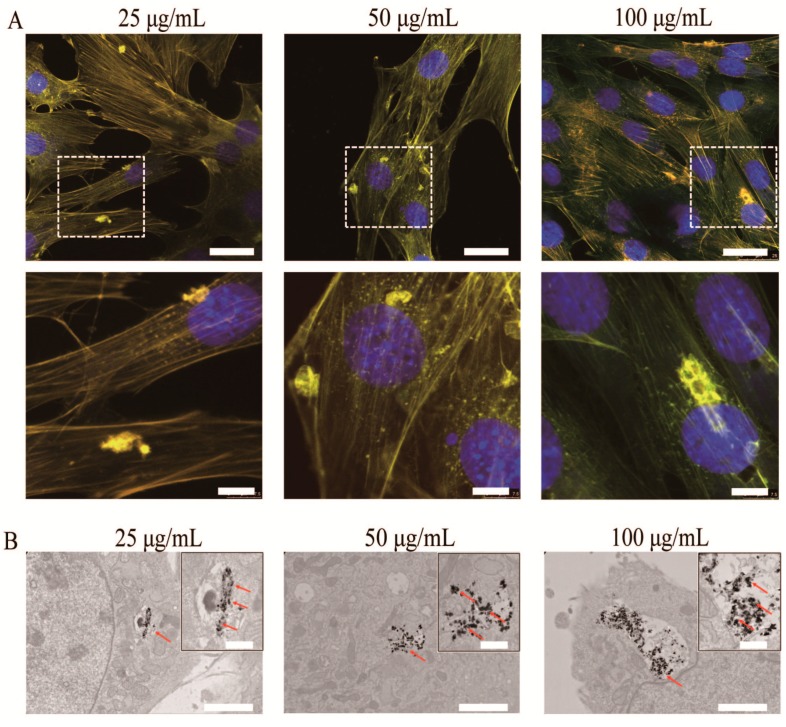

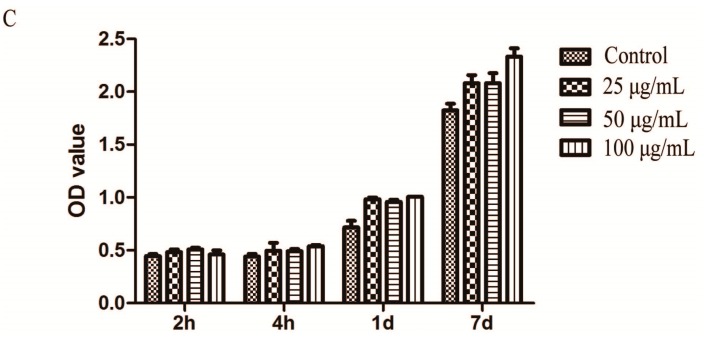
Biological reaction of MC3T3-E1 cells with different concentrations of Tb^3+^-doped hydroxyapatite (HA-Tb) nanorods (25, 50, and 100 μg/mL). (**A**) Representative fluorescent images of MC3T3-E1 cells after incubation with HA-Tb nanorods for 24 h at 37 °C. The bottom panel shows the magnified profiles indicated by the dashed white lines in the corresponding top panel. Cells were stained with fluorophore-labeled phalloidin and 4′,6-diamidino-2-phenylindole to visualize actin filaments and nuclei, respectively. The scale bars in the top and bottom panels are 25 and 7.5 μm, respectively; (**B**) Transmission electron microscopy images of MC3T3-E1 cells after incubation with HA-Tb nanorods. Red arrows indicate the uptake of HA-Tb nanorods into cells. The scale bar is 2 μm; the scale bar in the magnified field is 0.5 μm; (**C**) Results of Cell Counting Kit-8 assay for MC3T3-E1 cells after incubation with HA-Tb nanorods.

To explore the potential applications of upconversion HA-Tb fluorescent nanorods in the biomedical field, the uptake of HA-Tb nanorods into MC3T3-E1 cells was first evaluated. MC3T3-E1 cells were treated with 25, 50, or 100 μg/mL HA-Tb nanorods for 24 h at 37 °C. As illustrated in Figure 2A, the luminescence of the internalized HA in the intracellular cytoplasm was clearly observed under a fluorescent microscope, and the cells retained their normal morphology. The dark, electron-dense spots indicated by red arrows in the TEM images shown in Figure 2B correspond to HA-Tb nanoparticles rather than nanorods, confirming the strong uptake of HA-Tb in the cytoplasm of the MC3T3-E1 cells. The effect of HA-Tb nanorods on cellular viability was also investigated via Cell Counting Kit-8 (CCK-8) assay. The results show that the optical density (OD) continuously increased with increasing incubation time (Figure 2C).

### 2.3. Tissue Distribution of HA-Tb

**Figure 3:**
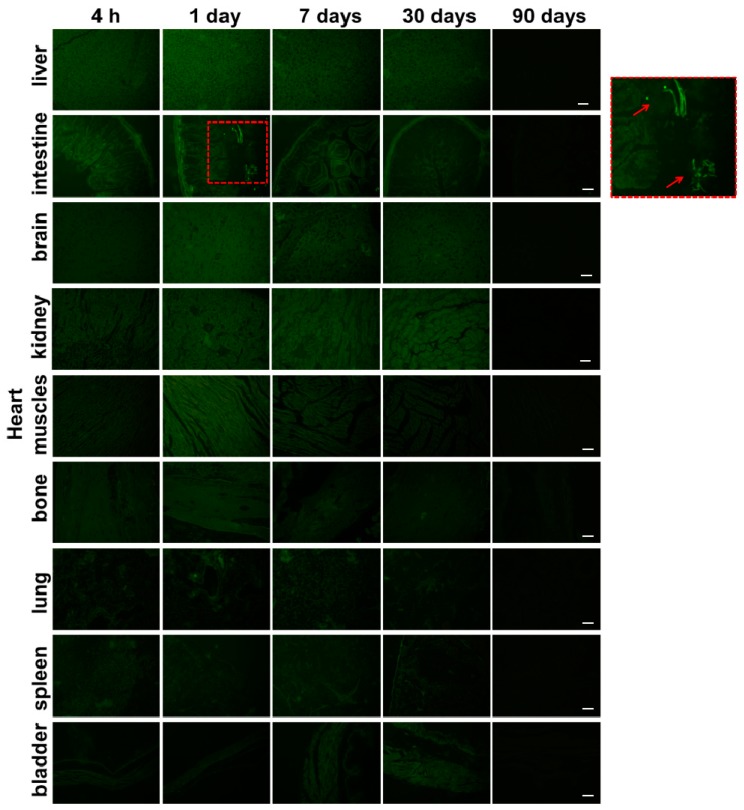
Tissue distribution of Tb^3+^-doped hydroxyapatite (HA-Tb) in various organs of rats at 4 h, one day, seven days, 30 days, and 90 days after the intraperitoneal injection of 50 mg/kg HA-Tb. See supplementary Figures S1 and S2 for the corresponding tissue distributions after intragastric and intravenous delivery. The right square shows the magnified profile indicated by the dashed red box in the second-from-top row. The red arrows indicate HA-Tb nanorods in the intestines. Scale bars are 20 μm.

**Figure 4:**
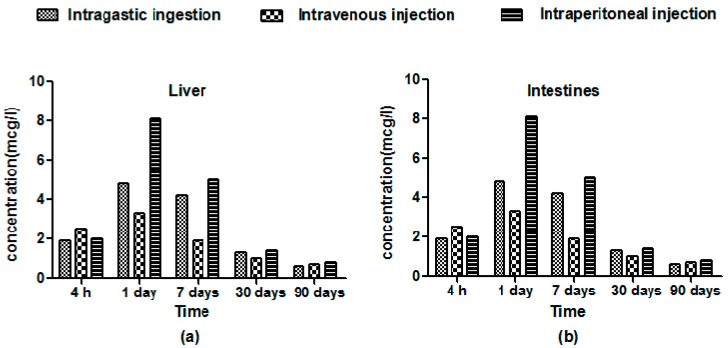

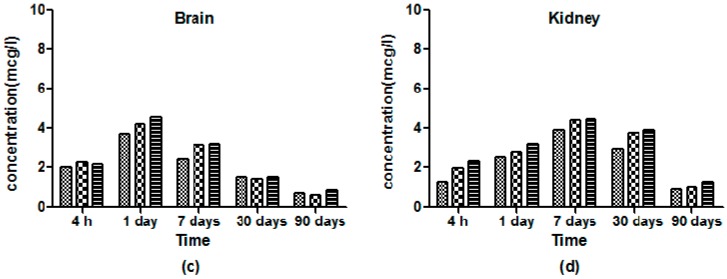
Mean Tb concentration-time profiles assayed by inductively-coupled plasma-mass spectrometry in the (**a**) liver; (**b**) intestines; (**c**) brain; and (**d**) kidney after the intragastric ingestion, intravenous injection and intraperitoneal injection of 25 mg/kg Tb^3+^-doped hydroxyapatite (HA-Tb) nanorods (*n* = 3).

The representative tissue distribution of HA-Tb was characterized by confocal laser scanning microscopy (CLSM) and measured by inductively-coupled plasma mass spectrometry (ICP-MS). The time-dependent variations in fluorescent intensity (Figure 3) and Tb concentration (Figure 4) showed similar trends for all three delivery methods. However, intraperitoneal injection yielded significantly higher fluorescent intensity and Tb concentration in experimental organs compared to intragastric and intravenous administration, especially in the liver and intestines. The Tb concentration in all organs was significantly different between the three administration methods (*p* < 0.05; Figure 4). At 4 h post-administration, the HA-Tb particles predominantly accumulated in the liver and intestines. Residual HA-Tb particles were mainly distributed in the intestines, liver, kidney, brain, and heart. Although lower levels of fluorescence were observed in the bone, lung, spleen, and bladder, they exceeded those of the control in all tissues. One day after administration, the uptake of HA-Tb in the intestines, liver, heart, muscles, liver, brain, and kidney increased, while no uptake was observed in the spleen. After the first day, uptake in most tissues continuously decreased, while uptake in the kidney and bladder increased continuously from 4 h to seven days. The lowest fluorescence intensity in all tissues was observed at 90 days post-administration.

### 2.4. Histology Toxicity

**Figure 5:**
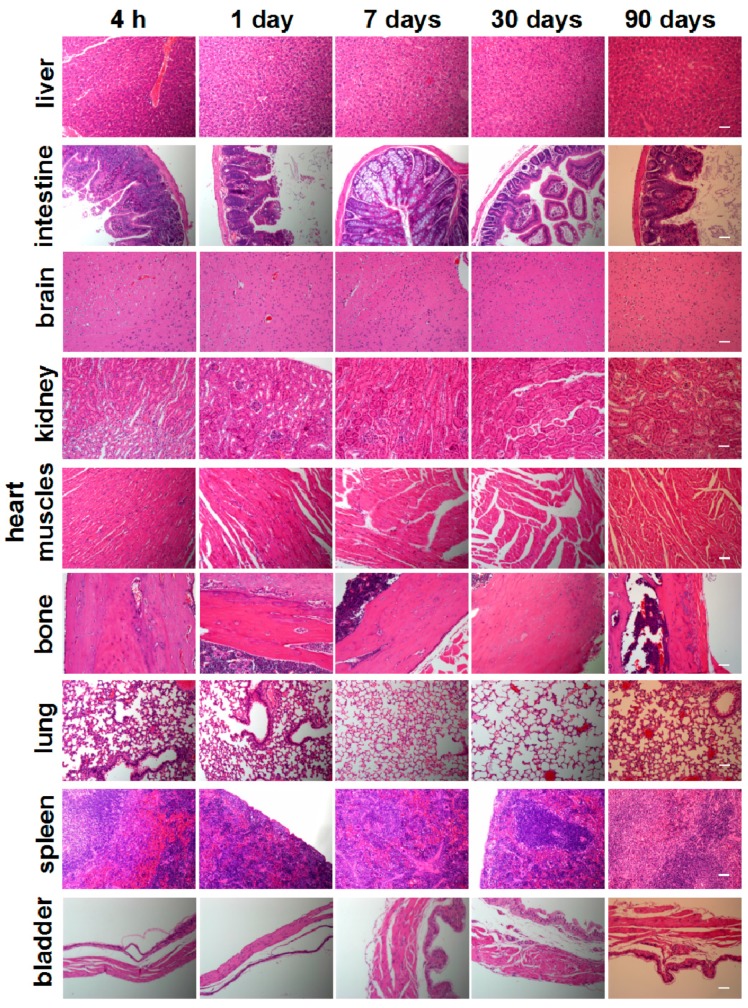
Histological analysis of various rat organs 4 h, one day, seven days, 30 days, and 90 days after intraperitoneal injection with 50 mg/kg Tb^3+^-doped hydroxyapatite (HA-Tb). The scale bar is 100 μm.

To investigate the in vivo toxicity of HA-Tb nanoparticles, we performed a histological analysis of organs to determine if HA-Tb or the byproducts of its degradation cause tissue damage, inflammation, or lesions (Figure 5). Hematoxylin and eosin staining showed that the morphologies of all organs of the experimental rats were comparable to those of the control group (Figure 5). No signs of necrosis were observed in any of the histological samples analyzed. The lung and spleen samples did not exhibit signs of inflammatory response, hyperplasia or fibrosis, and the cardiac muscles of heart samples did not exhibit hydropic degeneration. The hepatocytes from liver samples and the glomerulus structures in kidney samples were also normal.

## 3. Discussion

### 3.1. Uptake of HA-Tb Nanorods

The upconversion nanostructures proved to be favorable in cell labeling during cell-based therapy compared to organic luminophores and quantum dots because of their excellent anti-photobleaching capability, long excited-state lifetimes, and narrow emission spectra [17,18]. The uptake of HA-Tb nanorods into MC3T3-E1 cells was tracked. Luminescence of the internalized HA in the intracellular cytoplasm was clearly observed (Figure 2A), and the cells retained a normal morphology, demonstrating the excellent luminescence and cellular compatibility of HA-Tb. In a previous study, the size and shape of NM were shown to influence cellular uptake [19]. The higher internalizations of long (aspect ratio = 4), short (aspect ratio = 2), and spherical (aspect ratio = 1) nanoparticles were compared in human melanoma cells (A375). Endocytosis was observed for short nanoparticles, while the spherical nanoparticles were not endocytosed. The long nanoparticles were not totally endocytosed, resulting in the phenomenon of “frustrated phagocytosis,” which led to inflammation and, eventually, the formation of granulomas and pleural mesothelioma. The TEM images in Figure 2B confirm the strong uptake of HA-Tb in the cytoplasm of MC3T3-E1 cells through vesicle endocytosis, in accordance with previous reports [20,21,22,23,24]. The altered morphology of HA-Tb under intracellular conditions is attributed to the degradation in HA crystallinity caused by acidic endosomal trafficking.

Chor Yong Tay et al. used TR146 human oral buccal epithelial cells as an in vitro model to examine the cellular internalization, inflammatory response and cytotoxic effects of nano-TiO_2_ and nano-HA [25,26]. They found that exposure to nano-TiO_2_ and nano-HA resulted in an elevated level of reactive oxygen species level and increased cell contractility with significantly impaired wound healing capability, however, without any apparent cytotoxicity. In this study, the OD increased continuously with increasing HA-Tb incubation time (Figure 2C), indicating that HA-Tb had no significant cell toxicity at the examined doses. This result, which can be ascribed to the biocompatibility of HA and lanthanides, suggests the excellent biocompatibility of HA-Tb in intracellular cytoplasm. The cytotoxic linoleic acid molecules can be removed from HA nanorods by “cleaning” with NaOH-ethanol solution [27]. The resulting “clean” HA nanorods are expected to be even more biocompatible.

### 3.2. Effects of Delivery Method on Tissue Distribution

The observed accumulation in the liver, kidney, and lung can be ascribed to the clearance of nanoparticles from the blood by cells of the mononuclear phagocyte system [28,29]. The initial accumulation of HA-Tb in the brain demonstrated its ability to cross the blood–brain barrier, indicating potential applications in drug delivery for brain diseases. Higher levels of HA-Tb particles were distributed in the intestines and liver compared to in the spleen, suggesting that the intestines and liver are more important organs for HA-Tb metabolism than the spleen. The increased accumulation of HA-Tb in the kidney and bladder after seven days of blood circulation suggests that the urinary system contributes to the elimination of HA-Tb and that the intestines and liver fail to totally remove accumulated HA-Tb. These findings demonstrate that the intraperitoneal injection of HA-Tb is preferred to intragastric and intravenous delivery to facilitate tissue adsorption, and the excretion of HA-Tb involves the digestive, hematological, and urinary systems.

### 3.3. Histology Toxicity

Multiwalled carbon nanotubes were reported to contribute to granuloma formation in mice, raising serious concerns about the toxicity of nanomaterials [30,31,32]. In the present study, histological assessment did not reveal any histopathological abnormalities in organs. Overall, no acute or chronic toxicity related to the short-, intermediate-, or long-term treatment of HA-Tb was observed.

## 4. Materials and Methods

### 4.1. Preparation of HA-Tb Nanorods

Tb^3+^-doped HA nanorods were synthesized via a hydrothermal method as previously reported by Chen et al. [33]. Briefly, octadecylamine (0.5 g) was dissolved in 4 mL of oleic acid and 16 mL of ethanol, and an aqueous solution of Ca(NO_3_)_2_ (0.28 M, 7 mL) and Tb(NO_3_)_3_ (0.20 M, 2 mL) was added. The mixture was stirred for 3 min, and Na_3_PO_4_ (0.2 M, 7 mL) was then added to the solution. The mixture was stirred for an additional 10 min, sealed, and hydrothermally treated at a controlled temperature (160 °C) for 16 h. Finally, the samples were rinsed and lyophilized.

### 4.2. Cell Culture

MC3T3-E1, a mouse calvaria-derived cell line, was purchased from Cell Culture Center, Peking Union Medical College (Beijing, China). Cells were cultured in Dulbecco’s modified Eagle's medium (DMEM; Hyclone) supplemented with 10% fetal bovine serum (PAA, Coelbe, Germany), 100-IU/mL penicillin (Sigma, St. Louis, MO, USA), and 100-mg/mL streptomycin (Sigma) in an incubator (Sanyo, Toyota, Japan) with 5% CO_2_ at 37 °C and saturated humidity. The medium was changed every two to three days. After growing to 80% confluence, the MC3T3-E1 cells were digested by 0.25% trypsin (Sigma) and 0.02% ethylene diaminetetraacetic acid for further use.

#### 4.2.1. Bioimaging of HA-Tb Nanorods Uptaken in MC3T3-E1 Cells

The MC3T3-E1 cells were seeded onto 35-mm coverglass-bottom dishes and incubated in 2 mL of culture medium for 24 h. The culture medium was then removed, and the cells were incubated in 2 mL DMEM containing different doses of HA-Tb nanorods (25, 50, and 100 mg/mL) at 37 °C for an additional 24 h. After rinsing three times with phosphate-buffered saline (PBS) solution, the cells were fixed with 4% paraformaldehyde for 30 min. The stained cells were then rinsed twice with PBS before immediate observation by TEM and CLSM.

#### 4.2.2. Cell Viability Analysis

MC3T3-E1 cells were seeded in 96-well microplates at a density of 1 × 10^5^ cells mL^−1^ and cultured for 2 h, 4 h, one day, and seven days in media containing 25, 50, and 100 μg/mL HA-Tb nanorods. After incubation, cytotoxicity was measured by WST-8 assay using a CCK-8 cell proliferation kit (Dojindo Laboratories, Kamimashiki Gun, Kumamoto, Japan). A Varioskan Flash instrument (Bio-Rad 680, Microplate Master, Hercules, CA, USA) was used to measure absorbance at 450 nm. All experiments were carried out in triplicate, and three independent experiments were performed.

### 4.3. In Vivo Experiments

#### 4.3.1. Animals

Five-week-old male BALB/c mice were obtained from a specific pathogen-free colony at Charles River Japan Inc. and quarantined for 14 days before the study. A single dose of 100 μL HA-Tb suspension was administered to the mice at a dose of 50 mg/kg without anesthesia using one of three methods: oral administration, intravenous injection, or intraperitoneal injection. The animals were then harvested after 4 h, one day, seven days, 30 days, and 90 days. Six mice were harvested at each time point. The nanoparticular suspensions were checked for contamination by endotoxins using Limulus Amebocyte Lysate assay (Cambrex, Walkersville, MD, USA). Feed and water were available ad libitum except for during a one-night fast prior to harvest. At each time point, the animals were anesthetized with ether and weighed, and blood samples were collected from the abdominal vein for blood clearance analysis. The animals were then euthanized by exsanguination from the abdominal aorta, and tissues from different organs were harvested. The mice were handled in an accredited China Food and Drug Administration animal facility in accordance with the AAALAC International Animal Care Policies. The study protocol was reviewed and approved by the Animal Care and Use Committee of the National Institute of Toxicological Research, China Food and Drug Administration, China.

#### 4.3.2. CLSM and Histological Assessment

CLSM was used to determine the localization of the HA-Tb in the liver, spleen, kidneys, lung, heart, bone, intestine, bladder, and brain at each time point. Histological assessment was used to observe inflammatory reaction and tissue morphology. After the animals were sacrificed, all organs were fixed in 10% neutral-buffered formalin. The tissues were then processed and trimmed, embedded in paraffin, sectioned to a thickness of 3 μm, and stained with hematoxylin and eosin for microscopic examination.

#### 4.3.3. ICP-MS

The Tb content in the tissue samples was determined as an indirect indicator of the content of HA-Tb nanorods using ICP-MS at the School of Materials Science and Engineering, Tsinghua University. The samples were dried at 80 °C to constant weight and then digested in 65% HNO_3_ (5 mL/1 g wet tissue) at 90 °C for 90 min. The digested matter was diluted 100-fold, and Tb levels were measured.

### 4.4. Statistical Analysis

Results are expressed as mean ± standard deviation. Statistical significance was assessed with Student’s *t*-test or analysis of variance, as appropriate. The statistical significance for all tests was set at *p* < 0.05.

## 5. Conclusions

In summary, we characterized the in vitro cellular uptake and in vivo delivery-dependent histological behavior of HA-Tb nanoparticles. We evaluated the tissue distributions and toxicity of these nanoparticles after short-, intermediate- and long-term exposure. HA-Tb uptake into cells occurred via vesicle endocytosis. Excellent cell viability and no apparent changes in cell morphology were observed for HA-Tb nanoparticle concentrations ranging from 25 to 100 μg/mL. For in vivo delivery, intraperitoneal injection resulted in better bioavailability than intragastric and intravenous administration and facilitated the adsorption of HA-Tb by tissues. In all groups, the clearance of HA-Tb involved the digestive, hematological, and urinary systems. No apparent histopathological abnormalities or lesions related to treatment with HA-Tb were observed. These data suggest minimal in vitro and in vivo toxicity for all three modes of HA-Tb administration at the examined doses and time scales. The findings provide information for the design and development of fluorescent HA-based materials for biological applications.

## Supplementary Materials

The supplementary figures are available online.
