# Supplementary figures and images for: Cellular Uptake and Delivery-Dependent Effects of Tb3+-Doped Hydroxyapatite Nanorods
Source: Molecules. 2017 Jun 23;22(7):1043. doi: 10.3390/molecules22071043 (PMC6152145; doi:10.3390/molecules22071043)

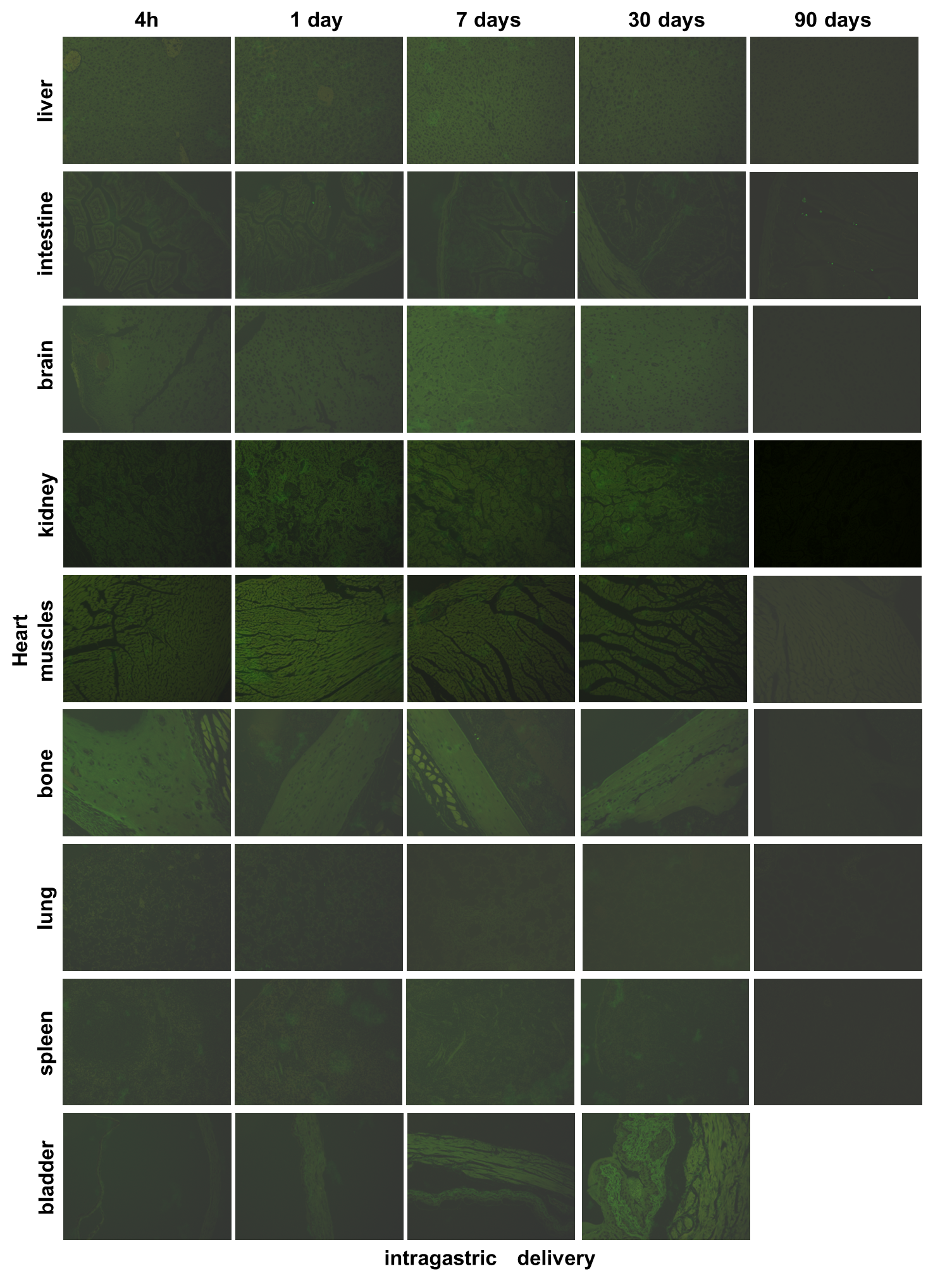

Supplement: Supplementary file 1 [file molecules-22-01043-s001.zip › supplementary/Supplementary Fig1.tif]

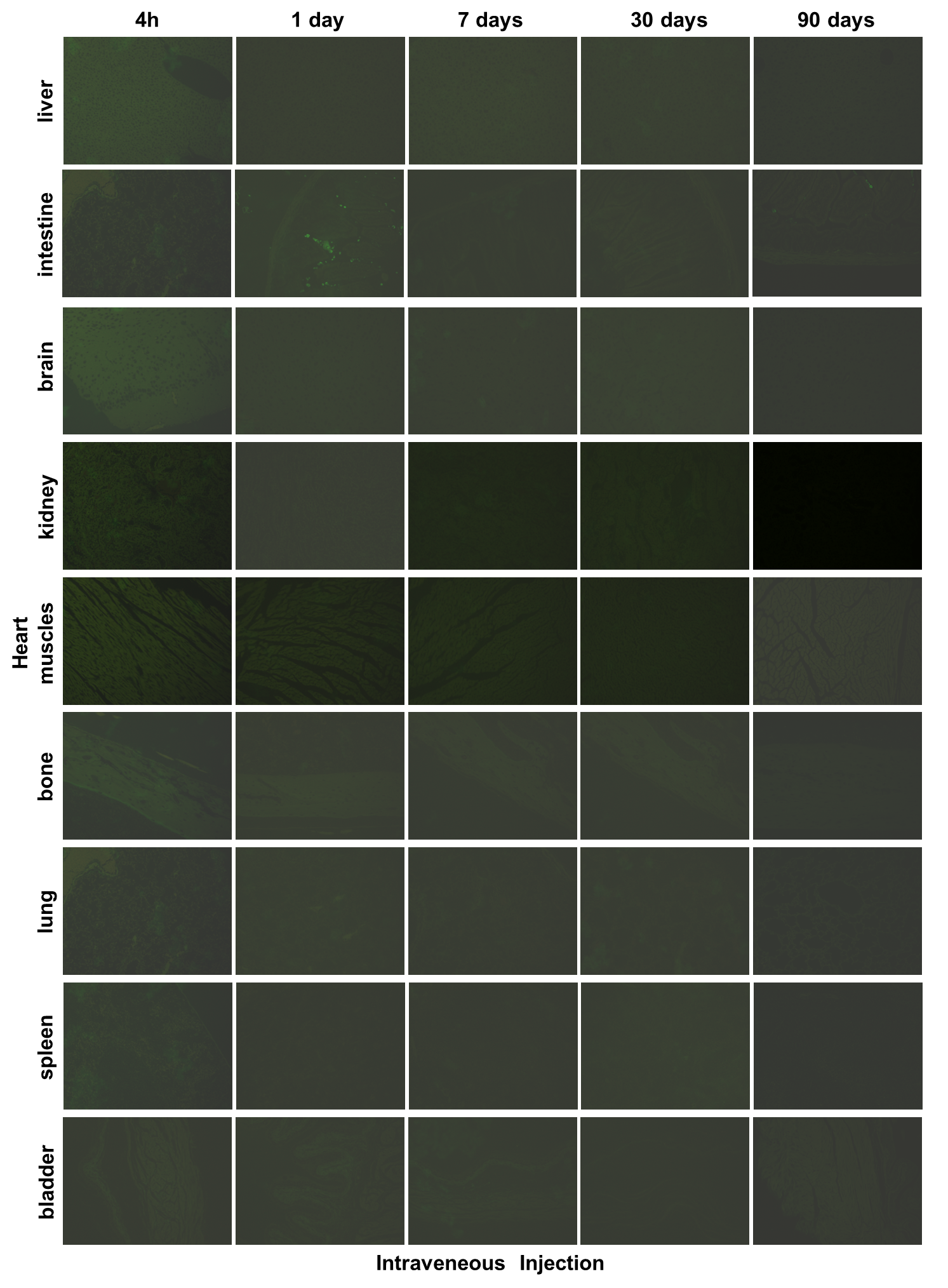

Supplement: Supplementary file 1 [file molecules-22-01043-s001.zip › supplementary/Supplementary Fig2.tif]
